# Polymer-Gated Bilayer Buccoadhesive Tablets for Biphasic Release of Indomethacin: Balancing Dissolution and Mucoadhesion

**DOI:** 10.3390/ph19060944

**Published:** 2026-06-15

**Authors:** Linhan Li, Jie Wang, Jie Xu, Jiaxin Li, Gang Jin

**Affiliations:** 1 School of Chemical and Pharmaceutical Engineering, Jilin University of Chemical Technology, Jilin 132022, China; 13844661417@163.com (L.L.); xu908613808@163.com (J.X.); lijiaxin202402@163.com (J.L.); 2 Party and Government Office, Jilin University of Chemical Technology, Jilin 132022, China; wangjie19861209@163.com

**Keywords:** bilayer buccal tablets, biphasic release, solid dispersions, polymer gelation, gastric-sparing delivery, mucoadhesion-dissolution balance, anti-solvent freeze-drying

## Abstract

**Objectives**: To address the critical limitations of current formulations that fail to simultaneously resolve indomethacin’s poor water solubility, susceptibility to gastric acid hydrolysis, and difficulty in balancing rapid onset with long-term sustained release, this study prepared solid dispersions via anti-solvent freeze-drying to improve drug dissolution, constructed oral buccoadhesive bilayer controlled-release tablets using direct powder compression, and elucidated the intrinsic relationships among polymer gel properties, swelling-erosion behavior, tablet integrity maintenance, and drug release mechanisms. **Methods**: Solid dispersions (SDs) were prepared by anti-solvent freeze-drying. Bilayer tablets (25 mg IND/tablet, 12.5 mg/layer) were fabricated via direct powder compression after optimizing disintegrants and polymer matrices. In vitro dissolution, surface pH, adhesion time, and adhesion strength were evaluated. **Results**: SDs enhanced dissolution by at least 30-fold in water and 2.4-fold at pH 6.8 within 2 h versus pure drug. Optimized bilayer tablets achieved 45% drug release at 20 min and 80% sustained release over 8 h, with surface pH of 6.8 ± 0.1, adhesion time of 8.3 ± 0.1 h, and adhesion strength of 57 ± 0.13 g. **Conclusions**: The physicochemical properties of polymeric excipients are critical for balancing drug release and mucoadhesion in buccal tablets. To achieve ideal controlled-release effects, in addition to focusing on the swelling and erosion characteristics of matrix-based tablets, the ability to maintain tablet integrity during dynamic dissolution must be further investigated, which is an essential factor for ensuring precisely modulated drug release. Meanwhile, when employing solid dispersions as solubilizing intermediates to prepare controlled-release formulations, the gelling properties of polymers in each formulation component should be fully considered to avoid incomplete disintegration and insufficient release at the initial dissolution stage.

## 1. Introduction

Indomethacin (IND), an indole-based non-steroidal anti-inflammatory drug (NSAID), was first discovered and synthesized in 1963 [1]. IND is widely used to treat inflammation, including arthritis and migraines, and prevent prostatitis, ophthalmic diseases, tumors, and postoperative inflammation [2,3]. IND primarily exerts anti-inflammatory and anti-tumor effects by inhibiting cyclooxygenase (COX-1/COX-2) activity and thereby reducing prostaglandin synthesis [4,5]. However, frequent use causes gastrointestinal injury and hepatorenal toxicity due to non-specific distribution, tissue accumulation, and prolonged prostaglandin inhibition [6]. Meanwhile, due to its low water solubility and high permeability, IND is classified as a Class II drug in the Biopharmaceutical Classification System (BCS) [7,8,9]. IND undergoes hydrolytic reactions in acidic or gastric acidic environments, and these factors collectively lead to a decrease in oral bioavailability [10,11]. To address the aforementioned issues, Xi et al. prepared IND-mesoporous silica-polymer solid dispersions using the solvent evaporation method, rendering the drug amorphous with 3-fold enhanced solubility and dissolution; mesoporous silica confinement and carrier hydrogen bonding inhibited recrystallization and improved stability [12]. To address drug hydrolysis in gastric acid environments, Asghar et al. incorporated acrylic resins into a xanthan gum matrix to prepare IND colon-targeted tablets. This design significantly inhibited drug hydrolysis in gastric acid while achieving colon-specific delivery and reducing gastrointestinal irritation. Meanwhile, xanthan gum, as a hydrophilic gel matrix material, rapidly swells upon hydration to form a dense gel layer, achieving sustained release through retarding drug diffusion. Additionally, the acrylic resins in the tablets could construct a pH-responsive controlled-release system, ultimately achieving precise colon-targeted controlled release [13]. In the field of sustained-release (SR) formulation research, researchers prepared IND microencapsulated SR tablets to modulate drug release behavior through microencapsulation technology. This approach achieved smooth and sustained release over 12 h without obvious drug burst release, while the final release amount reached 94.2%, accomplishing favorable SR effects [14]. Currently, numerous studies have focused on drug solubilization methods, hydrolysis behavior in gastric environments, and the preparation of SR tablets, but overlook temperature and solvent residue effects on stability and release; systematic data on gastric instability during solubilization and delayed onset from conventional SR formulations remain scarce.

To overcome the negative effects of poor water solubility, researchers have explored a series of techniques, including eutectic formation, salt synthesis, particle size reduction, self-emulsifying drug delivery systems (SEDDS), and SDs [15,16,17]. SDs are the most widely applied and promising strategy [18], dispersing insoluble drugs in hydrophilic polymers and converting crystals to amorphous forms to enhance solubility [19]. After solubilization through SD technology, drugs can be further formulated into conventional dosage forms, such as tablets, capsules, and suppositories. The core technological direction for changing the frequency of drug administration is to develop long-acting formulations. At the same time, it can also achieve effective therapeutic effects by preparing sustained-release and controlled-release formulations while reducing the frequency of drug administration. In response to the problem of drug hydrolysis in gastric acidic environments, current technologies have achieved ideal drug release effects while avoiding first-pass effects through buccal membrane administration and pH-sensitive coatings.

Owing to its advantages such as easy ingestion, suitability for self-medication, accurate dosage, flexible and controllable dosing regimens, high patient compliance, and low risk of administration difficulties, oral administration has become the most common and preferred route for drug delivery [20]. Tablets dominate controlled-release systems for tunable release and stability, including single/multilayer, membrane, microcapsule, and osmotic pump designs. For BCS Class II drugs, researchers have systematically investigated the effects of hydroxypropyl methylcellulose (HPMC) with different molecular weights on the diffusion and dissolution coupling mechanisms of drugs. By selecting polymers with different molecular weights to form the skeleton, the pore-forming behavior can be differentiated to achieve rational drug release [21]. Researchers have converted the insoluble drug diclofenac into a multilayer skeleton tablet. The inner core was first prepared via wet granulation and then wrapped with a rapid release layer to form a multilayer skeleton tablet structure. Simultaneously, an acrylic resin ammonio methacrylate copolymer (RL/RS) blend was selected as the key excipient to regulate the drug release rate, ensuring the harmonious balance between rapid and sustained release. This design solves the problem of ordinary formulations, where it is difficult to balance rapid onset and long-term maintenance [22]. Meanwhile, in the development of bilayer tablet formulations, mature research reports have also been published for tramadol, a BCS Class I drug with high solubility and high permeability. Researchers have altered the drug dissolution curve by adjusting the excipient and Tramadol content in the immediate release (IR)and SR layers of the tablets. The optimized IR layer was released for 15 min, whereas the SR layer was slowly released over 10 h. Compared to commercially available tablets, this bilayer tablet has a longer half-life and reduces the frequency of daily administration [23].

However, although the abovementioned administration routes can achieve ideal controlled release effects, they still cannot solve the problem of controlled release of unstable drugs in the gastric juice environment. Therefore, with the continuous development of drug delivery systems, buccal drug delivery has attracted extensive attention owing to its advantages in effectively circumventing the adverse effects of the gastric environment on drugs and enabling a controllable initial drug release rate [24,25]. It bypasses first-pass metabolism, irritation, and degradation for faster onset and higher bioavailability [26]. For example, researchers have introduced bioadhesive polymers into SR tablets of tizanidine hydrochloride and delivered them through the buccal mucosa, successfully avoiding first-pass metabolism of the drug in the liver and hydrolysis in gastric juice during oral administration. This indicates that the rational modification and application of polymers are important factors in oral buccal membrane drug delivery systems.

One of the most critical factors for achieving the desired controlled-release efficacy of diverse drug types in oral and buccal drug delivery systems is the rational application of polymer materials. Commonly used slow and controlled-release polymers can be divided into two categories: natural and synthetic polymers. These polymers serve as core carriers or skeletal materials, achieving long-term drug delivery by regulating drug diffusion and dissolution rates. Subramanian et al. used sodium alginate (SA) as a hydrophilic matrix material for controlled-release baclofen matrix tablets, which regulated drug release by forming a gel barrier when it met water. The tablets also exhibit good mechanical properties and storage stability [27]. Rosario-Meléndez et al. developed a biodegradable polyurethane synthetic polymer as a skeleton material for SR analgesic drug formulations that combines drug activity and skeleton support function. The polymer achieves long-term sustained release of drugs through self-degradation, providing a new formula solution for long-term pain relief and overcoming the single functional limitation of traditional skeleton materials as carriers [28]. Therefore, the rational selection and use of polymers plays an important role in the development of tablets.

In response to the key issues of IND, including poor water solubility, easy hydrolysis in acidic environments, low oral bioavailability, and the difficulty for conventional formulations to balance rapid onset and long-term sustained release, this study aims to develop an oral mucoadhesive bilayer controlled-release tablet. IND SDs were prepared via an anti-solvent freeze-drying method to improve its dissolution performance. By rationally selecting gelling polymers and optimizing the formulations of IR and SR layers, the intrinsic relationships among carrier gelation properties, swelling-erosion behaviors, and drug release were elucidated. Ultimately, a bilayer formulation with rapid initial release, stable long-term drug delivery, and suitable mucoadhesion was obtained, providing a scientific basis and technical reference for the rational design of oral mucoadhesive controlled-release formulations of poorly water-soluble drugs.

## 2. Results

### 2.1. Analysis of the Solubility of IND

From the solubility data of IND listed in Table 1 in different media, it could be seen that the solubility of IND in water is extremely low, only 8.8 ± 0.01 μg/mL. Therefore, it was classified as a water-insoluble drug [29]. However, under pH 6.8 conditions, the solubility of IND significantly increased to 176 ± 0.01 μg/mL, showing a certain degree of pH dependence. The primary reason is that IND is a carboxylic acid-type acidic compound with a dissociation constant (pK_a_ ≈ 4.5). Consequently, under acidic conditions, the drug exists predominantly in its neutral molecular form, resulting in extremely low aqueous solubility. When the medium is water (pH 5.5) and pH 6.8 buffer, the pH exceeds the pK_a_ value. As the pH increases, the carboxyl group undergoes progressive ionization with a corresponding increase in the degree of dissociation, and its solubility increases accordingly. Notably, the solubility of IND at pH 1.2 could not be determined due to its rapid hydrolysis in highly acidic media [30]. High-performance liquid chromatography (HPLC) analysis further confirmed that under the same chromatographic conditions, no characteristic peak of IND was detected in the drug solution at pH 1.2. This directly confirms the chemical instability of IND under gastric acidic conditions, which is the core scientific rationale driving the development of buccal delivery formulations to avoid acid-induced degradation. In addition, it was found that sodium lauryl sulfate (SLS), cetyltrimethylammonium bromide (CTAB) and Tween-80 significantly increased the solubility of IND, with solubility values reaching 308 ± 0.05 μg/mL, 598 ± 0.05 μg/mL, and 211 ± 0.01 μg/mL, respectively. This was mainly because the concentration of the surfactants used in the solubility experiments was much higher than the critical micelle concentration (CMC). At this concentration, the surfactants self-assemble into micelles that can encapsulate water-insoluble drugs in their cores and significantly increase drug solubility [31].

Research has found that, except for polyvinylpyrrolidone K30 (PVP K30), the solubility of IND in different carrier aqueous solutions improved to a certain extent. This phenomenon was mainly due to the good water solubility of these carrier materials and the neutral pH of the carrier solution, which enhanced the solubility of the drug to some extent. Although PVP K30 has good water solubility, the weak acidity of its aqueous solution was the main factor limiting its improvement of drug solubility.

To further explore the factors affecting the solubility of IND, the solubility results and pH changes of the IND excipient solutions before and after equilibration were analysed. The results show that the solubility of IND is pH-dependent and usually increases with increasing pH [32]. The solubility of IND in the three surfactant solutions was significantly higher than that at pH 6.8, whereas the pH values of the CTAB and Tween-80 solutions before and after shaking were both below pH 6.8. These results indicate that the formation of surfactant micelles could partially offset the decrease in IND solubility caused by low pH values. Mechanistically, micellar solubilization overrides pH effects, highlighting that solubilization strategies must account for both pH dependence and carrier–drug intermolecular interactions. Therefore, micelle formation plays a dominant role in improving drug solubility [33].

### 2.2. Dissolution Studies

**Figure 1:**
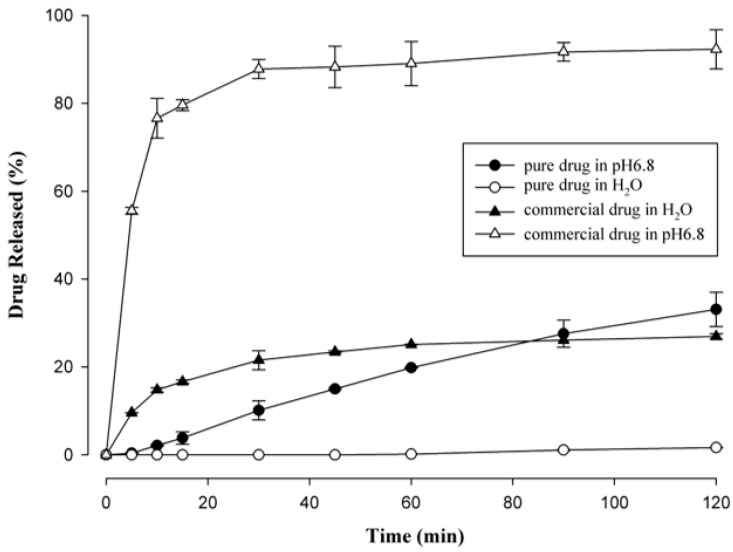
Dissolution curves of IND and commercial drug in 900 mL deionized water and pH 6.8. Values are expressed as mean ± SD, n = 3 in each group.

From the in vitro drug dissolution curves of IND and commercial drugs in water (Figure 1), it can be seen that the dissolution of pure drugs and commercial drugs in water is very low. The dissolution within 2 h only reached 1% ± 0.1% and 27% ± 0.1%, while in pH 6.8, it reached 33% ± 0.1% and 92% ± 0.1% (*p* < 0.05). Consistent with the solubility data, the dissolution of IND showed a pH dependence, with higher values at pH 6.8, which is also reflected in commercial drugs.

**Figure 2:**
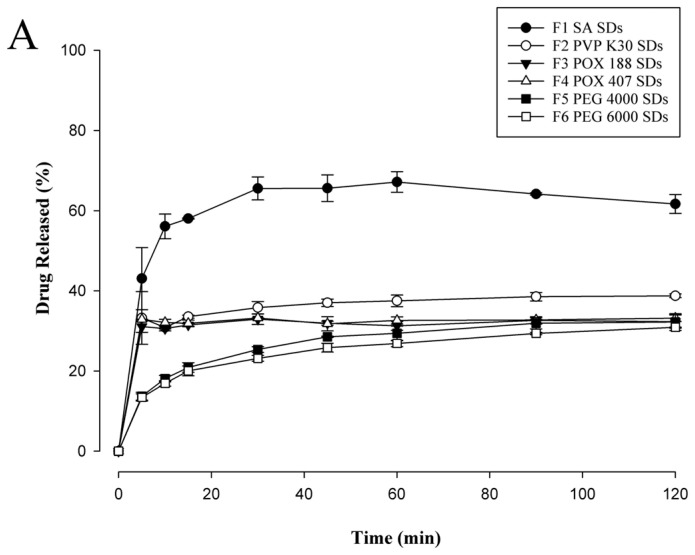

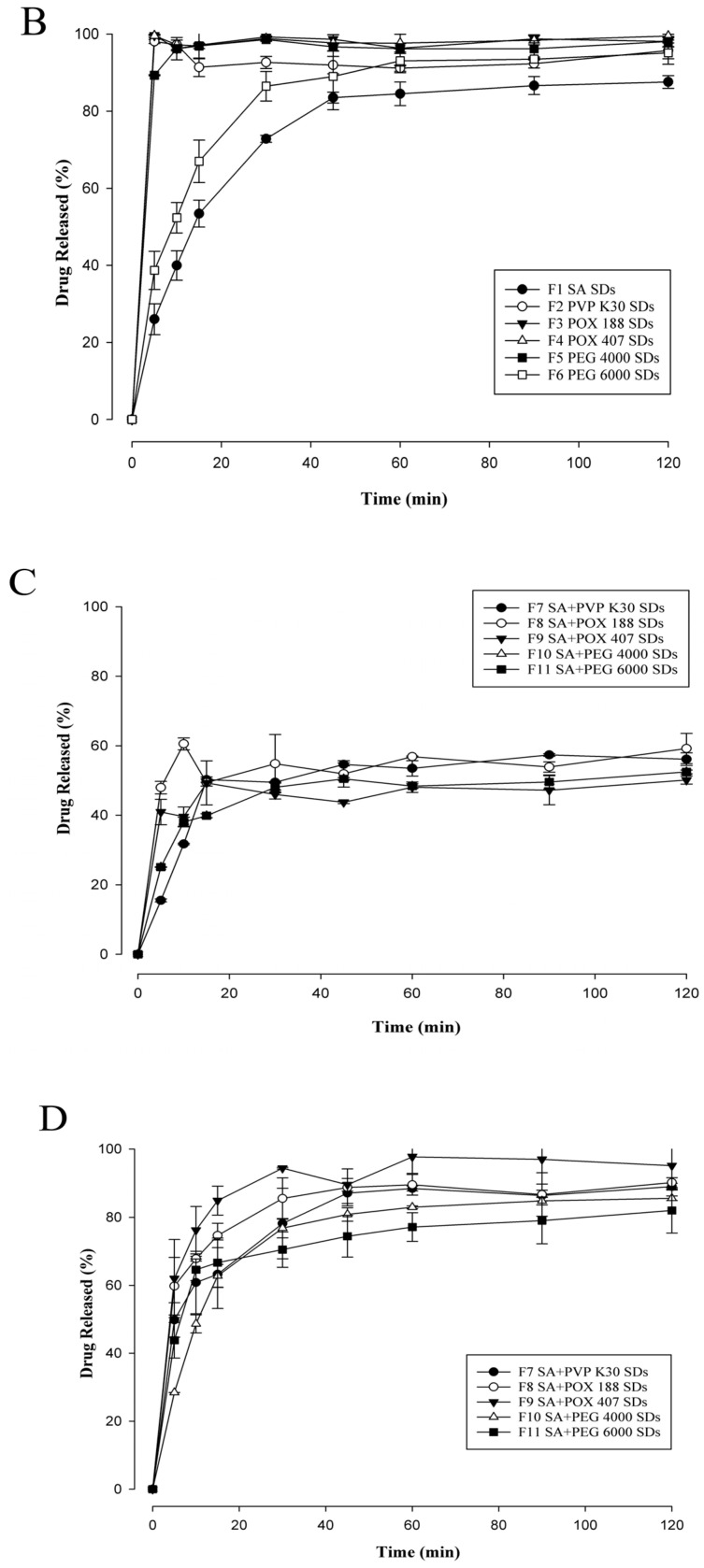
Dissolution curves of IND-SDs prepared with different carriers in 900 mL of water (**A**,**C**) and pH 6.8 (**B**,**D**). SA SDs, sodium alginate solid dispersions; PVP K30 SDs, polyvinylpyrrolidone K30 solid dispersions; poloxamer 188 SDs, POX 188 SDs; poloxamer 407 SDs, POX 407; polyethylene glycol 4000, PEG 4000; polyethylene glycol 6000, PEG 6000. Values are expressed as mean ± SD, n = 3 in each group.

In this study, we aimed to develop a bilayer-controlled-release tablet. To meet design requirements, the primary technical key is to improve the solubility of IND to achieve the initial rapid release of the drug. To further evaluate the potential of these carriers in improving drug dissolution, IND-SDs were prepared and the in vitro dissolution behavior was evaluated. As shown in Figure 2, all SDs prepared from the carrier materials showed a certain degree of improved IND dissolution in deionized water and pH 6.8 (*p* < 0.05).

The dissolution curves of SDs prepared using different carrier materials in water in Figure 2A indicate that, except for SA, which can increase to 64% ± 1%, all other polymers show an increasing trend, but can only reach around 30% (*p* < 0.001, decreased by 34%). This is mainly because, in the dissolution experiment, the formation of the SA gel has a certain wrapping effect on drug particles. The SA gel slows down the speed of initial burst release to the medium, thus inhibiting the dissolution decline caused by drug recrystallization to a certain extent, and ensuring the initial drug release rate [34]. Mechanistically, SA gelation creates a diffusion barrier that kinetically suppresses amorphous-to-crystalline reversion, a key instability pathway for SDs, thereby preserving dissolution enhancement. Figure 2B shows the dissolution curves of the SDs prepared with different carriers at pH 6.8. IND dissolves relatively easily in high-pH media because of its pH dependence. Therefore, the dissolution of all SDs was significantly higher than that of the pure drugs and significantly higher than the dissolution of SDs in water. SDs prepared with poloxamer (POX) 188, POX 407, PVP K30, and polyethylene glycol (PEG) 4000 achieved 100% in vitro dissolution after 5 min (*p* < 0.05). Notably, the dissolution of SA-SDs at pH 6.8 is lower than that of SDs prepared with other polymers (*p* < 0.05). This is because the encapsulation of SA on drug particles, to a certain extent, impedes contact between drug particles and the dissolution medium, resulting in a low initial drug release rate [35]. Figure 2C,D show the dissolution curves of SDs prepared with SA and different carrier materials in water and pH 6.8. Dissolution in water demonstrated that when SA was co-formulated with other polymers as carriers for preparing SDs, the dissolution of drugs in water was 15% higher than that of SDs prepared using only a single polymer (*p* < 0.05, increased 15%). This phenomenon shows that the gel network can slow the speed of drug exposure to the medium to a certain extent, thus slowing the occurrence of recrystallization and improving drug dissolution [36]. Therefore, when using SA to prepare SDs, it should be noted that the formation of a polymer gel slows the contact rate between drugs and water by wrapping the drugs, which, to some extent, affects the release rate at the initial stage of dissolution. Simultaneously, using SD technology, most crystalline drugs are transformed into amorphous forms that are easily recrystallized. SA can wrap drugs by forming gels that can slow the rate of drug recrystallization to a certain extent when suddenly exposed to the medium.

**Figure 3:**
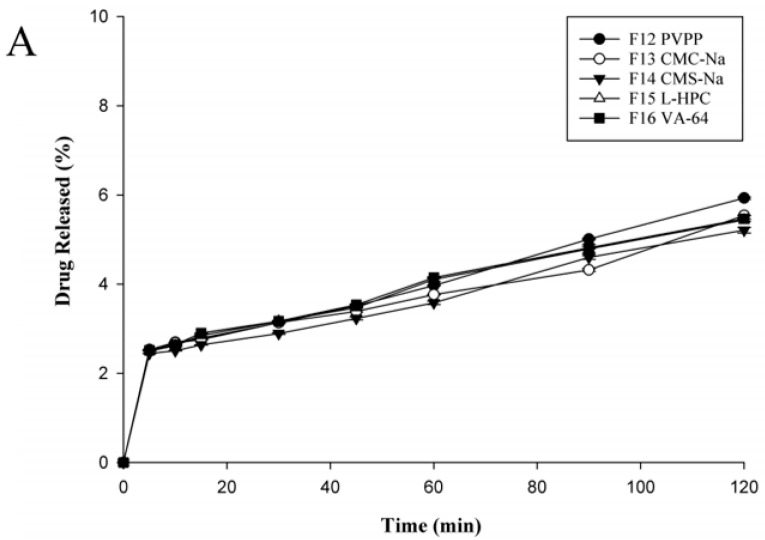

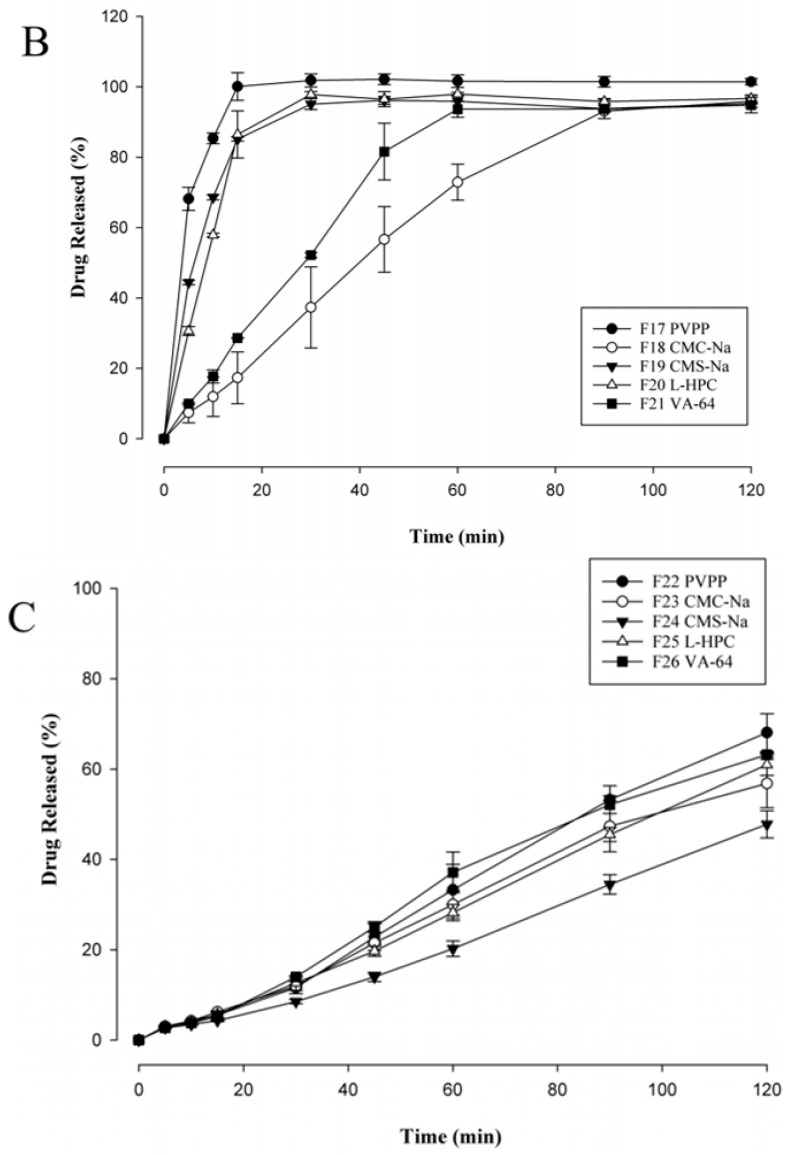
Dissolution curves of IR layers prepared with SA SDs (**A**), PVP K30 SDs (**B**) and SA + PVP K30 SDs (**C**) in 900 mL pH 6.8. Crosslinked polyvinylpyrrolidone, PVPP; sodium carboxymethyl starch-Na, CMS-Na; sodium carboxymethyl cellulose-Na, CMC-Na; low-substituted hydroxypropyl cellulose, L-HPC; vinylpyrrolidone-vinyl acetate copolymer-64, VA-64. Values are expressed as mean ± SD, n = 3 in each group.

Figure 3 shows the in vitro dissolution curves of the IR layers prepared with different SDs and disintegrants at pH 6.8. As shown in Figure 3A, the IR layer based on SA SDs exhibits extremely low in vitro drug release, reaching only 5% ± 0.1% over 2 h (*p* < 0.05). This is in sharp contrast to the SA-SDs powder, which dissolved 80% under the same conditions (Figure 2B). The main reason for this difference is that the formation of SA gel reduces the rate of media entering the core [37]. The disintegrant in the tablet loses its ability to disintegrate rapidly, whereas the drug in the core is difficult to dissolve.

Figure 3B shows that the IR layer prepared with the PVP K30 SDs released over 90% of the drug over 2 h, and there was no significant difference in dissolution compared to the SD powder (*p* > 0.05). Owing to the weak gelling ability of PVP K30, when the tablet came into contact with the medium, the dissolution medium quickly passed through the surface of the tablet to reach the core. Simultaneously, the disintegrant is rapidly absorbed and expanded, accelerating the dissolution of the core drug, thereby achieving the desired release performance of the drug. Notably, all disintegrants achieved a dissolution rate of 85% of the drug over 2 h, and polyvinylpolypyrrolidone (PVPP), owing to its high coefficient of expansion, had a significantly higher initial dissolution rate than the other disintegrants (*p* < 0.05) [38]. Mechanistically, high expansion of PVPP generates rapid matrix disruption, maximizing medium access and aligning with the IR layer’s core goal of rapid onset. However, the low initial dissolution rate of tablets prepared with sodium carboxymethyl cellulose (CMC-Na) as a disintegrant was due to the gel characteristics of CMC-Na. This gelation creates a barrier that hinders the penetration of the dissolution medium into the tablet core, consequently leading to the retardation of drug release (*p* < 0.05) [39,40]. Figure 3C shows the dissolution curve of the IR layer prepared using SA + PVP K30 SDs. Although the formulation showed a slow initial dissolution rate, its final drug release exceeded 40% over 2 h, which was significantly higher than the release in Figure 3A (*p* < 0.001, increased 35%). Compared with Figure 3A, the reduction in the amount of SA in SDs reduced the strength of the gel formed when encountering water, and to some extent, increased drug release. Therefore, the influence of the gel characteristics of each component on tablet release should be considered when optimizing tablet formulations. This dose-dependent gel effect establishes a quantitative mechanism for balancing rapid release and stability, directly supporting the study’s rational formulation design rationale.

**Figure 4:**
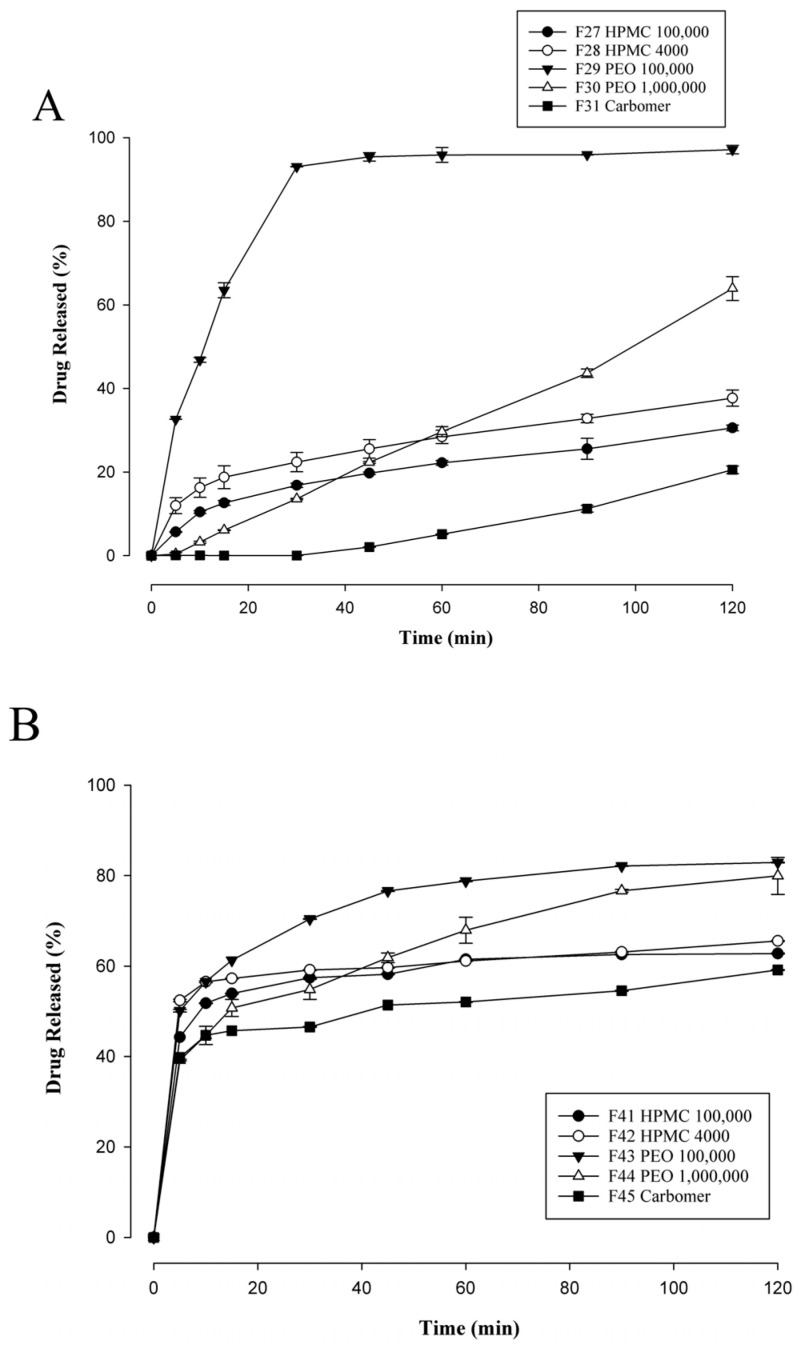
Dissolution curves of SR layers (**A**) and bilayer tablets (**B**) in 900 mL pH 6.8 for 2 h. Hydroxypropyl methyl cellulose 100,000, HPMC 100,000; hydroxypropyl methyl cellulose 4000, HPMC 4000; polyethylene oxide 1,000,000, PEO 1,000,000; polyethylene oxide 100,000, PEO 100,000; carbomer. Values are expressed as mean ± SD, n = 3 in each group.

Polymer-based sustained-release systems are widely used for preparing controlled-release tablets [41]. The core principle of this system is to regulate drug release by introducing hydrophilic polymers with gel characteristics into the tablet skeleton. The drug release rate of the system is predominantly determined by the properties and viscosity of the polymer gel as well as the overall swelling and erosion behaviors of the tablet [42]. Figure 4 shows the dissolution of the SR layers prepared with PVP K30 SDs and various SR polymers at pH 6.8, as well as the bilayer tablets composed of these SR layers and the optimal IR layer based on PVP K30 SDs also at pH 6.8. As shown in Figure 4A, all polymers exhibited effective sustained-release effects, except for two different molecular weights of polyethylene oxide (PEO), with less than 30% of the drug released within 2 h. However, PEO 100,000 failed to exert an SR effect and achieved over 90% dissolution within 30 min (*p* < 0.05). This is mainly because the hydrated layer formed by the low-molecular-weight PEO is thin and weak, making the tablet vulnerable to rapid erosion [43]. Although PEO 1,000,000 showed good initial SR effects, its erosion rate increased over time, which may have led to a rapid drug release in the middle and later stages. As shown in the figure, drug release reached 60% within 2 h, which made it challenging to ensure stable and long-term drug release in the later stages. The SR layers formulated with the carbomer and HPMC exhibited relatively stable SR behavior, with dissolution changes ranging from 10 to 20% and 15 to 25%, respectively, over 2 h (*p* < 0.05). The primary reason for this stable SR performance was that both polymers possessed high network structure strength and viscosity. After the tablet comes in contact with the medium, the thick swelling layer formed on the surface maintains the integrity of the tablet structure for a long time, effectively preventing the sudden release of drugs. The dissolution curves obtained when these SR layers were compressed together with the optimal IR layer into bilayer tablets are shown in Figure 4B. The dissolution of the bilayer tablets in pH 6.8 medium is slightly lower than the theoretical additive value of the corresponding IR and SR layers at each time point (*p* < 0.05). This was mainly because the formation of the polymer gel in the SR layer slightly blocked the drug release rate in the IR layer when the tablets were in contact with the medium.

**Figure 5:**
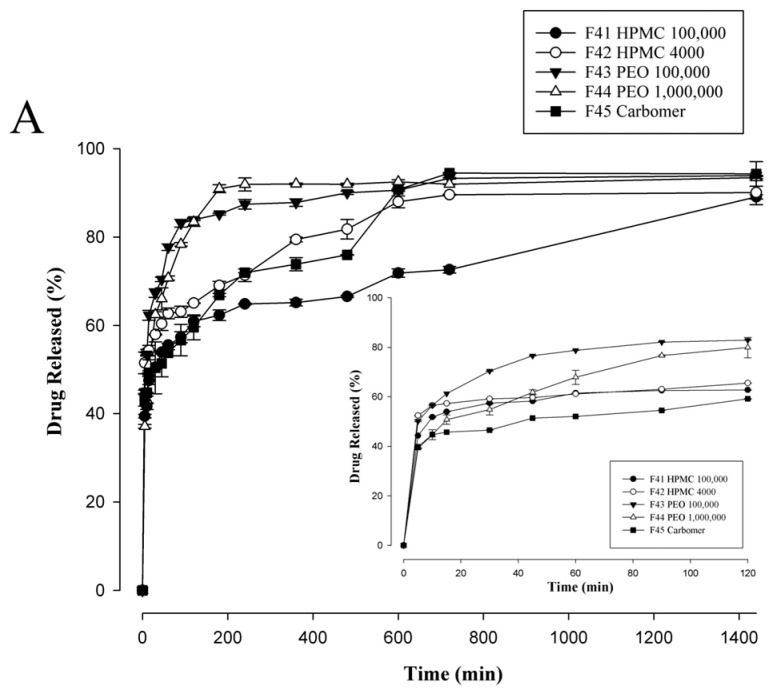

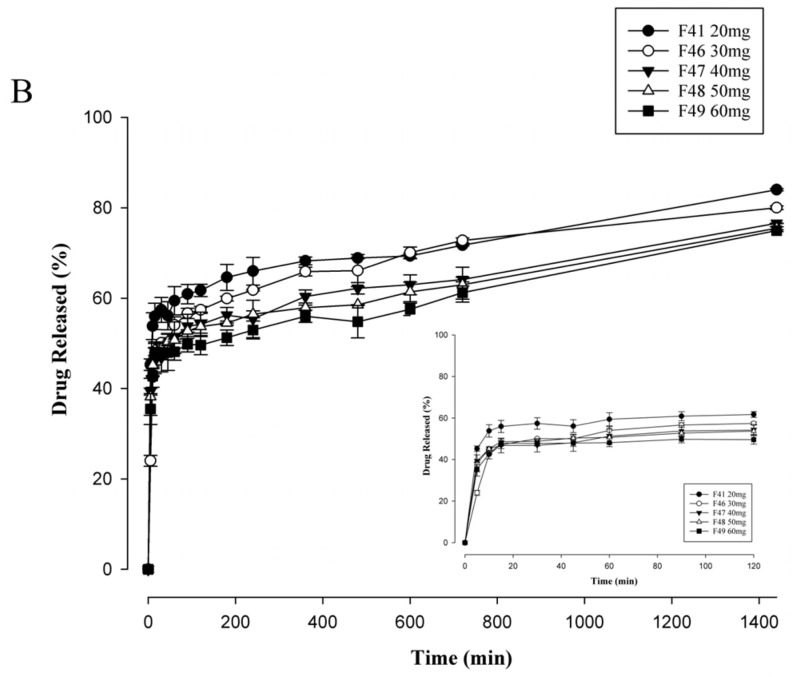
Dissolution curves of bilayer tablets prepared with different polymers (**A**) and different amounts of HPMC 100,000 (**B**) in 900 mL pH 6.8 for 2 h. Hydroxypropyl methyl cellulose 100,000, HPMC 100,000; hydroxypropyl methyl cellulose 4000, HPMC 4000; polyethylene oxide 1,000,000, PEO 1,000,000; polyethylene oxide 100,000, PEO 100,000; carbomer. Values are expressed as mean ± SD, n = 3 in each group.

To investigate the long-term SR effect of bilayer controlled-release tablets, the drug release of different polymers as skeleton materials in a medium of pH 6.8 was studied. As shown in Figure 5A, the bilayer tablets prepared with PEO of different molecular weights failed to effectively delay drug release, owing to the weak matrix structure and high erodibility. In contrast, bilayer tablets prepared with HPMC showed a relatively rapid drug release during the first 2 h, followed by a sustained stable release over the following 22 h. When comparing the use of HPMC with different molecular weights, because HPMC 100,000 has a higher swollen network strength and viscosity than HPMC 4000, its slow-release performance is better than that of HPMC 4000 (*p* < 0.05) [44]. In addition, while ensuring the integrity of the tablets, it guarantees a better drug release effect. The bilayer tablets formulated with carbomer exhibited a favorable controlled release effect during the first 8 h. However, owing to the high water absorption of carbomer, a large amount of medium continuously penetrated the tablet matrix between 480 and 600 min. This excessive infiltration damages the structural integrity of the tablet matrix, ultimately leading to a sudden drug release [42].

The effect of the bilayer tablets formulated with varying amounts of HPMC 100,000 on drug release is shown in Figure 5B. As the amount of polymer in the tablet increased, the drug release at each time point gradually decreased. This is primarily because HPMC 100,000, upon hydration, simultaneously impedes medium penetration into the tablet core and further enhances the resistance to drug outward diffusion. To ensure that the final release amount of the tablet met the standards and minimized the impact of the SR layer polymer on the drug release from the IR layer, 20 mg of HPMC 100,000 was ultimately selected as the optimal dosage for the SR layer in the bilayer tablet.

**Figure 6:**
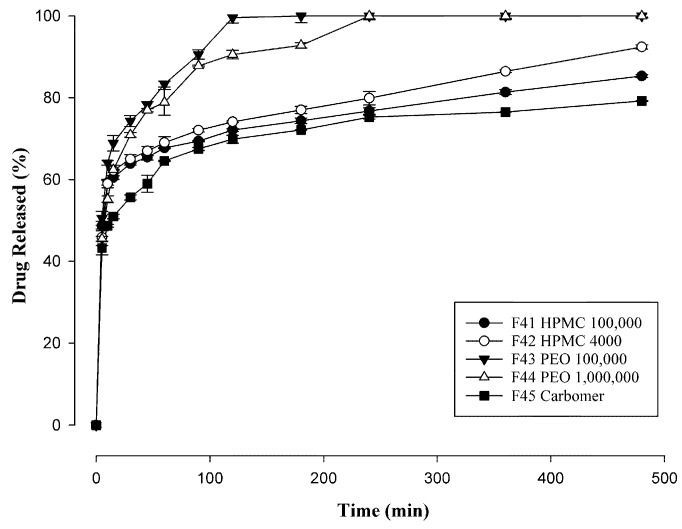
Dissolution curves of bilayer tablets prepared with different polymers in 250 mL pH 6.8. Hydroxypropyl methyl cellulose 100,000, HPMC 100,000; hydroxypropyl methyl cellulose 4000, HPMC 4000; polyethylene oxide 1,000,000, PEO 1,000,000; polyethylene oxide 100,000, PEO 100,000; carbomer. Values are expressed as mean ± SD, n = 3 in each group.

Considering that IND is prone to hydrolysis and the formation of byproducts at pH < 2 or >10, the resulting byproducts lack relevant pharmacological activity, significantly reducing the therapeutic effect of the drug [30]. Therefore, avoiding drug exposure to acidic environments can be achieved by coating the tablets or administering them through oral buccal membranes, both of which can effectively alleviate the negative effects caused by drug hydrolysis. Considering the aforementioned negative effects and referring to the established protocol for simulating saliva dissolution studies, the drug release behavior of this bilayer tablet was evaluated in 250 mL pH 6.8 medium [45]. As shown in Figure 6, drug release from the tablet for 8 h followed the same trend as in the previous 900 mL of pH 6.8 medium. Except for PEO, the bilayer tablets showed a rapid initial drug release of 45%, followed by a sustained stable release, ultimately reaching 80%. In summary, bilayer tablets can be administered through the oral buccal membrane, which not only avoids adverse reactions related to the gastric acid environment but also overcomes the disadvantage of delayed initial release caused by tablet coating, thereby achieving the goal of improving bioavailability.

In summary, the mechanisms by which the main carrier materials used in the preparation of SDs and the main sustained-release matrix materials employed in tablet manufacturing regulate dissolution behavior can be primarily categorized into the following three aspects: (i) gel-forming capability; (ii) swelling and erosion characteristics; and (iii) hydration network structural strength. When carrier materials with gel-forming properties are used to prepare SDs (Figure 2A,C), the gel encapsulates the drug, mitigating the phenomenon of sudden drug exposure to the medium and the consequent recrystallization, thereby slowing the initial drug release rate; however, this gel encapsulation effect also reduces the ultimate drug release amount (Figure 2B). When combined with PVP K30, which possesses weaker gel-forming properties, as a composite carrier material, the overall drug release rate and final release amount are collectively affected. When employed as matrix materials for tablet preparation, the use of polymeric polymers can modulate the swelling rate and matrix erosion behavior of tablets upon hydration, thereby influencing the overall tablet release profile (Figure 4A,B). Meanwhile, the type and molecular weight of the polymers used in tablet preparation regulate the hydration network structural strength, which in turn governs the matrix erosion process and drug release rate (Figure 5A,B). In conclusion, the gel-forming ability, swelling and erosion characteristics, and hydration network strength of the carriers directly determine the dissolution rate, extent of drug release, and curve stability of the formulation.

### 2.3. Analysis of the Physical and Chemical Properties of Bilayer Tablets

Table 2 shows the physical and chemical properties of bilayer tablets prepared using different polymers. The weight, hardness, thickness, diameter and drug content of the bilayer tablets prepared for each formulation were within the range of 130 ± 0.23 mg–130 ± 0.55 mg, 49 ± 0.4 N–49.91 ± 0.6 N, 2.5 ± 0.01 mm–2.5 ± 0.02 mm, 9.8 ± 0.01 mm–9.8 ± 0.02 mm, 98.95 ± 0.2–101.2 ± 0.2%, indicating that the use of powder direct compression technology has good stability. These consistent mechanical and uniformity parameters are mechanistically linked to polymer–particle interactions during compression, ensuring reproducible performance and supporting the study’s goal of robust buccal delivery systems. The surface pH value of the tablet was 6.72 ± 0.2–6.80 ± 0.1, which is within the acceptable range of oral mucosa (5.5–7.0) and does not cause significant discomfort.

### 2.4. Analysis of Swelling, Erosion, Disintegration Time and Mucosal Adhesion Characteristics of Bilayer Tablets

Through swelling and erosion tests, the swelling and erosion rates of PEO with different molecular weights in Table 3 showed significant differences. The swelling rate of high-molecular-weight PEO 1,000,000 is 1041 ± 12%, much higher than the swelling rate of low-molecular-weight PEO 100,000, which is 890 ± 10% (*p* < 0.001). Owing to its high swelling property, the preparation showed high matrix structural strength and reduced the erosion rate by 16 percentage points over 30 min (*p* < 0.001). This phenomenon is primarily attributed to the semicrystalline nature of PEO. As the molecular weight increased, the proportion of amorphous regions expanded. After the tablet comes into contact with the medium, the polymer chain in the amorphous region absorbs the medium and expands to form a viscous matrix structure [46]. Mechanistically, higher molecular weight increases amorphous content, enhancing water uptake and gel crosslinking, which directly correlates with improved structural integrity and sustained release. Therefore, there are differences in swelling and erosion between high- and low-molecular-weight PEO, and high-molecular-weight PEO is more likely to maintain tablet integrity.

**Figure 7:**
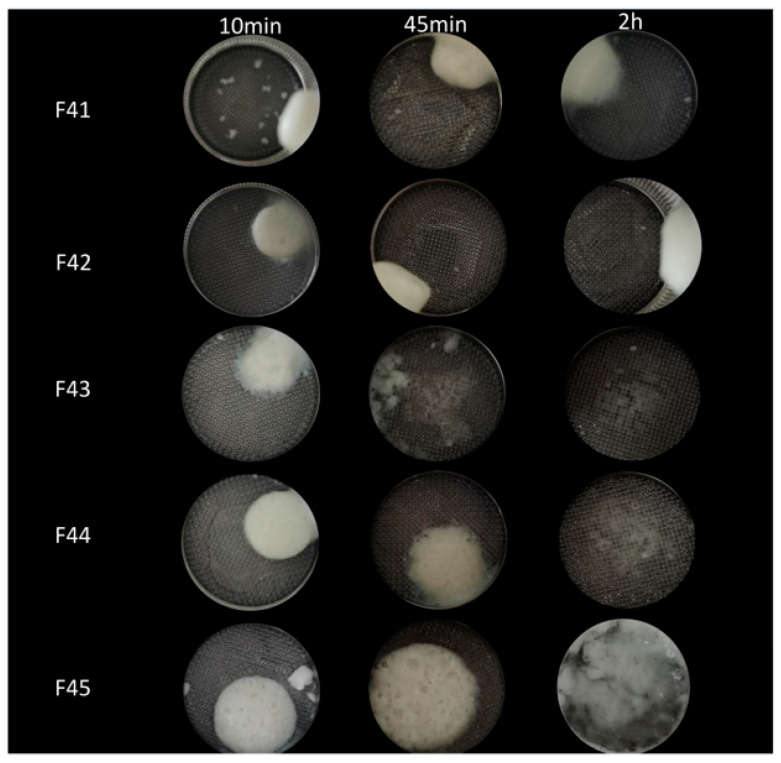
The images of the bilayer tablets with different formulations as a function of time during the dissolution test. F41—HPMC 100,000; F42—HPMC 4000; F43—PEO 1,000,000; F44—PEO-1,000,000; F45—Carbomer.

The disintegration times in Table 2 also confirm this conclusion, with PEO 1,000,000 having a disintegration time of 152 min, which is significantly longer than that of PEO 100,000 (*p* < 0.001). Unlike other polymers, swelling and erosion data for high-molecular-weight PEO and low-molecular-weight PEO after 120 min are not presented. The main reason for this is that, compared with other polymers, PEO has a relatively weaker hydration ability, which cannot effectively delay the disintegration and erosion of tablets. The morphological changes of formulation F44 and F43 based on high- and low-molecular-weight PEO at three time points in Figure 7 further confirm this conclusion, with the tablets in the basket completely disappearing after 2 h.

Comparing the swelling data of five polymers used for preparing bilayer tablets, carbomer had the highest swelling rate, reaching 8633 ± 20% (*p* < 0.001). Because of its extremely low crystallinity and almost completely amorphous structural characteristics, the amorphous region is the main area for tablet water absorption and expansion, resulting in a much higher swelling rate than that of other polymers [47]. Notably, the swelling rate of the carbomer exhibits a steady upward trend during the first 480 min. Although the swelling rate doubled between 480 and 600 min, the integrity of the carbomer-based tablets was not maintained throughout the swelling erosion test. As shown in the dissolution curve in Figure 5A, drug release increased from 75 ± 1% to 90 ± 1% between 480 and 600 min, further confirming the poor ability of the formulation to maintain tablet structural integrity during dissolution. As shown in Figure 7, the tablet almost lost its structural integrity after 2 h; however, because of its excellent swelling and self-height viscosity, it adhered to the dissolution basket in the form of a gel after contact with the medium. This phenomenon resulted in an apparent, rather than actual, low erosion rate due to the residual gel mass. Mechanistically, excessive swelling of carbomer leads to matrix loosening and catastrophic erosion, explaining its late-stage burst release and limiting its use as a sole SR matrix. Therefore, when carbomers alone are used as the matrix skeleton material for bilayer tablets, it is difficult to maintain the overall structural integrity of the dosage form, thereby increasing the risk of sudden drug release during dissolution [42]. Similarly, owing to its high gel performance and swelling, it can be used in combination with other polymers to achieve better sustained and controlled release effects [48].

Compared to PEO, which erodes rapidly, and carbomers, which cannot maintain the integrity of the tablet structure, the hydroxyl and methoxy groups on the HPMC polymer chains have a superior ability to form a strong hydrogen-bonding network. The hydrogen bond network was more stable than the weak hydrogen bond network derived from the physical entanglement of the PEO chain and the gel network formed by the swelling and cross-linking of the carbomer chain [49,50]. This stronger intermolecular bonding is the fundamental mechanism underlying HPMC’s unique balance of swelling, erosion resistance, and mucoadhesion. In addition, HPMC demonstrated superior ability to maintain the integrity of the bilayer tablet structure. The morphologies of the tablets at different time points in Figure 7 further confirmed that they maintained good structural integrity during testing.

At the same time, mucosal adhesion characteristics in Table 2 indicated that HPMC 100,000 exhibits the longest adhesion time (8.3 ± 0.1 h) and the highest adhesion force (57 ± 0.13 g) (*p* < 0.05). These results confirm that the optimal formulation of bilayer tablets has good structural integrity and sufficient viscosity, ensuring that controlled-release tablets formulated with the main adhesive polymer can meet the requirements of oral adhesion. In conclusion, HPMC 100,000 was identified as the optimal polymer for adhesion and controlled drug release. This is due to its excellent matrix-forming properties and high swelling capacity, which not only ensures the structural integrity of the bilayer tablet during the entire drug release process, but can also accurately adjust the drug release and has good mucosal adhesion.

### 2.5. FTIR Analysis

**Figure 8:**
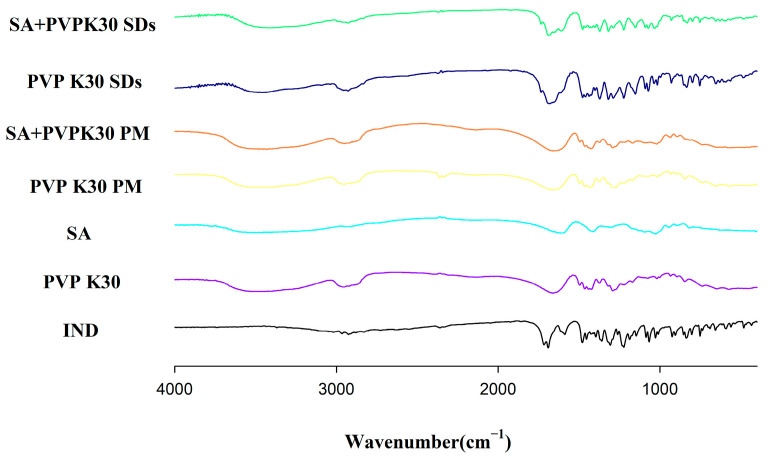
FTIR of IND, PVP K30, SA, PVP K30 PM, SA + PVP K30 PM, PVP K30 SDs and SA + PVP K30 SDs.

The FTIR spectra of pure IND, PVP K30, SA, PM, and SDs are shown in Figure 8. IND exhibited characteristic peaks at 1709 cm^−1^, corresponding to the carboxylic acid carbonyl group (C=O); at 3420 cm^−1^, attributed to (N–H) stretching vibrations; and at 1583 cm^−1^, assigned to the benzoyl amide carbonyl group (C=O) [51]. PVP K30 displayed a carbonyl stretching vibration peak at 1665 cm^−1^ (C=O), whereas SA exhibits a characteristic carbonyl absorption peak (C=O) in the range of 1700–1720 cm^−1^, and a hydroxyl (-OH) stretching peak in the range of 3200–3600 cm^−1^ [52,53]. For the preparation of SDs, the characteristic peaks that are easily affected by intermolecular interactions are concentrated in the amide carbonyl peak of PVP K30, the carboxylic carbonyl peak of IND, and the hydroxyl stretching vibration peak of SA. However, comparative FTIR analysis showed no significant changes in the characteristic peaks of IND in PM and SDs. This indicates that there is no intermolecular chemical interaction between the components in SDs and PM.

### 2.6. PXRD Analysis

**Figure 9:**
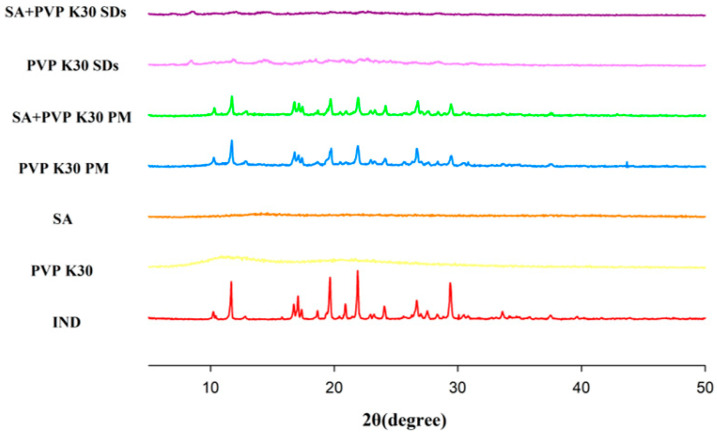
PXRD of IND, PVP K30, SA, PVP K30 PM, SA + PVP K30 PM, PVP K30 SDs and SA + PVP K30 SDs.

The PXRD results are shown in Figure 9. IND exhibited a series of distinct crystal diffraction peaks in the range of 10–30°, indicating that IND is highly crystalline. PVP K30 and SA, which were used as carrier materials, exhibited no obvious crystal diffraction peaks, indicating that PVP K30 and SA exist in an amorphous form. PXRD patterns of all PM are only the superposition of characteristic diffraction peaks of each component, which indicates that physical mixing has not changed the original morphology of each component. In contrast, the characteristic peaks in SDs do not exhibit a large number of sharp diffraction peaks within the range of 15–30°, unlike the active pharmaceutical ingredient IND. However, weak characteristic peaks of IND can still be observed at 12°, 20° and 22°. These results demonstrated that the two SDs prepared by this method exhibited a partial transformation of the IND crystal form into an amorphous form, which is conducive to improving the water solubility of the drug.

### 2.7. FE-SEM Analysis

**Figure 10:**
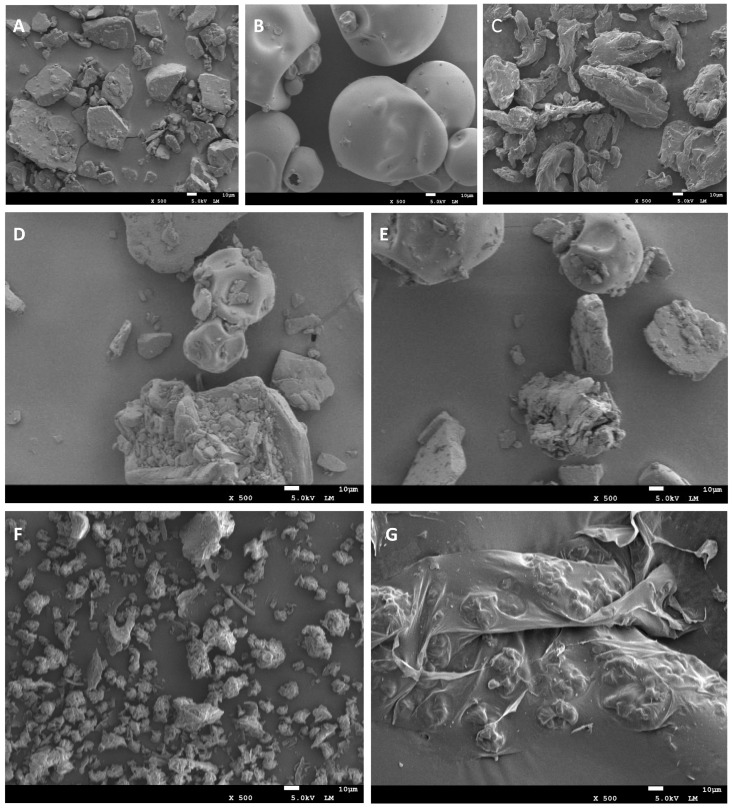
FE-SEM of (**A**) IND; (**B**) PVP K30; (**C**) SA; (**D**) PVP K30 PM; (**E**) SA + PVP K30 PM; (**F**) PVP K30 SDs and (**G**) SA + PVP K30 SDs.

FE-SEM images of pure IND, PVP K30, SA, PM, and SDs are shown in Figure 10. IND (Figure 10A) presented irregular shapes with different sizes and rough surfaces, PVP K30 (Figure 10B) presented a similar smooth spherical shape, and SA (Figure 10C) presented irregular bodies with smooth surfaces but slightly visible wrinkles. For PM (Figure 10D,E) only the original forms of each component were superimposed, indicating that the original form of the drug in PM did not change. In contrast, PVP K30 SDs (Figure 10F) existed as particles with a narrower particle size distribution and smoother surface morphology. No obvious original monomer morphologies of IND and PVP K30 were observed in the figure, indicating that the SDs prepared by this method altered the crystal morphology of the drug to some extent. When SA was incorporated as a composite carrier into the PVP K30 to prepare SDs (Figure 10G), the dried products did not exhibit the inherent form of a single component. In contrast, owing to the gel-forming characteristics of SA, each component was integrated into a whole, showing the rough surface and uneven distribution of irregular bodies.

## 3. Materials and Methods

### 3.1. Materials

IND was provided by Aladdin Biochemical Technology Co., Ltd. (Shanghai, China). Polyvinylpyrrolidone K30 (PVP K30) was obtained from Shanghai McLean Biochemical Co., Ltd. (Shanghai, China). Polyethylene oxide (PEO 100,000, PEO 1,000,000), hydroxypropyl methylcellulose (HPMC 4000, HPMC 100,000), poloxamer (POX 188, POX 407), polyethylene glycol (PEG 4000, PEG 6000), sodium alginate (SA) and carbomer were purchased from Shanghai Aladdin Biochemical Science and Technology (Shanghai, China). HPLC grade acetonitrile was supplied by Tianjin Damao Chemical Reagent Factory (Tianjin, China). All other chemicals were of analytical grade and were used without further purification.

### 3.2. Solubility Study

Deionized water, phosphate buffer (pH 1.2/6.8), and various 1% (*w*/*v*) auxiliary aqueous solutions were selected as media for the solubility experiments. Excess IND was added to a 10 mL centrifuge tube containing different media and mixed well, and the pH was measured. Then, it was placed in a water bath at 37 °C and continuously shaken at 100 rpm for 24 h. The pH was measured again after shaking and the supernatant in the sample solution was filtered through a 0.45 micron microporous filter, diluted with mobile phase and analyzed by HPLC. All experiments were conducted in triplicate (n = 3).

### 3.3. Preparation of IND-SDs

The IND-SDs were prepared using an anti-solvent freeze-drying method. PVP K30 (25 mg) was completely dissolved in 4.5 mL deionized water to form a transparent PVP K30 aqueous solution. Simultaneously, 25 mg of IND was dissolved in 1.5 mL of absolute ethanol to obtain a clear drug solution, which was then added to the PVP K30 solution. After continuous stirring at 500 rpm at room temperature (25 ± 2 °C) for 0.5 h, the mixture was pre-frozen at −83 °C for 24 h and then freeze-dried at −80 °C under vacuum (10–30 Pa) for 24 h to obtain IND-SDs. The composition of the formula for preparing IND-SDs is presented in Table 4.

### 3.4. Formulation and Preparation of IND Bilayer Tablets

The IR and SR layers based on the best SDs were prepared using the direct powder compression method. Each ingredient was thoroughly ground and passed through a 40-mesh sieve. For the IR layer, according to its formula, SDs, disintegrant, and filler were fully mixed using a mixer, and magnesium stearate was added and mixed again. The powder direct compression method used to compress the IR tablets and the composition of the IR layer are listed in Table 5. Similarly, the SR layer was processed using the aforementioned IR layer method according to its formula (Table 6) and pressed into a tablet. For the bilayer tablet, the SR layer was pre-pressed, and then the powder of the IR layer was added. Table 7 shows the formulation of the bilayer tablets. During tablet compression, the compression force for the IR layer was 12 kN, and that for the SR layer was 10 kN. For the bilayer tablets, the pre-compression force for the IR layer was 3 kN, and the main compression force for the SR layer was 8 kN. The hardness of the tablets was controlled within the range of 49–51 N (n = 3).

### 3.5. In Vitro Release Studies

The USP dissolution II paddle method was used to study the drug release in vitro, and the drug release of IND (25 mg), IND-SDs (25 mg IND), the IR layer (12.5 mg IND), the SR layer (12.5 mg IND) and the bilayer tablet (25 mg IND) was measured at 50 rpm and 37 ± 0.5 °C. The in vitro release of IND, IND-SDs, and commercial drugs was studied in 900 mL water and pH 6.8 for 2 h. The development of bilayer tablets based on IND-SDs aimed to evaluate drug release in a simulated oral saliva environment in vitro and after oral administration through formulation optimization and reasonable application of polymers. Therefore, for the in vitro release test of bilayer tablets, the release conditions at 8 h and 24 h in 250 or 900 mL with pH 6.8 were mainly investigated. At each designated sample collection time point, 3 mL of the sample was withdrawn, and an equal volume of the culture medium was added to maintain a constant volume during dissolution. The collected samples should be filtered through a 0.45 μm PVDF filter and diluted with mobile phase for HPLC analysis. The 2 h sampling time points were set at 5, 10, 15, 30, 45, 60, 90, and 120 min, and the 24 h sampling time points were collected at 5, 10, 15, 30, 45, 60, 90, 120, 180, 240, 360, 480, 600, 720, and 1440 min (n = 3).

### 3.6. Disintegration Time Study

Six tablets were randomly selected from each formulation and placed in the basket of a disintegrator (SY-6 DN, Shanghai Huanghai Drug Inspection Instrument Co., Ltd., Shanghai, China) containing 900 mL of pH 6.8 phosphate buffer. Then, the instrument was run at a pH of 6.8 and a temperature of 37 ± 0.5 °C until all of the tablet fragments passed through the sieve completely. The average disintegration time of each formulation was also calculated (n = 3).

### 3.7. Hydrodynamic Behaviors of the Bilayer Tablet

In this study, the water swelling rate (*WU%*) and erosion rate (*ES%*) were measured to evaluate the effects of different polymers on the release behavior of the bilayer tablets. Each tablet was weighed with continuous stirring at the speed of 50 rpm, and its initial weight (*IW*) was recorded, and then placed in pH 6.8 medium at 37 ± 0.5 °C. At each predetermined time point, the tablet was taken out and the excess surface moisture was carefully removed using filter paper to obtain the wet weight (*WW*) of the tablet, and then dried in an oven at 35 °C until a constant dry weight (*DW*) was reached. The following formula was used to calculate the tablet *WU* (%) and *ES* (%) at each specific time (n = 3).

(1) $$WU\left(\%\right)=\frac{WW-DW}{DW}\times 100\%$$

(2) $$ES\left(\%\right)=\frac{IW-DW}{IW}\times 100\%$$

### 3.8. Uniformity Evaluation of Tablet Hardness, Weight, Thickness, Diameter, Friability, Drug Content and Surface pH Value

Ten tablets were randomly selected from each formulation, and their weight, diameter, and thickness were assessed. An electronic balance was used to accurately weigh the tablets and the average value was determined (HZK-FA 110, HUAZHI, Fuzhou, China). The diameter and thickness of the tablets were measured using digital calipers to ensure that the tablet thickness was within 5% of the theoretical value. The hardness of the tablets (SY-6 DN, China) was evaluated using a hardness tester. Ten tablets were selected from each formula and the average hardness value (N) was calculated. To measure friability, each batch (n = 10) was weighed and the initial total weight was recorded. The sample was then placed on a roller and rotated 100 times. After removing the tablets, the total weight was measured again, and the corresponding formula was used to calculate the friability of the tablets.

(3) $$Friability\left(\%\right)=\frac{Initail\;weight-Final\;weight}{Initail\;weight}\times 100\%$$

The ground tablet powder (1 mg) was accurately weighed and dissolved in a volumetric flask containing 10 mL of the mobile phase (n = 3). A mixer and ultrasonic treatment were used to pass the obtained clear drug solution through a 0.45 μm microporous filter membrane and then the drug content was determined by HPLC. Before pH analysis, the surface of the tablet with a pH of 6.8 was contacted for a period of time, and then the electrode of the pH meter (PHS-3 E pH meter, INSEA Scientific, Shanghai, China) was placed on the surface of the tablets for 2 min to measure the pH value of the microenvironment [54]. Each sample was analyzed in triplicates (n = 3).

### 3.9. Ex Vivo Mucoadhesive Strength

All animal experiments were performed with protocols approved by the Institutional Animal Care and Use Committee of Jilin University of Chemical Technology (Approval No. 20251017, Jilin, China). An improved balance method was used to evaluate the adhesion strength of mucous membranes in vitro. The porcine oral mucosa tissue (1 × 1 cm), soaked in pH 6.8 for 1 h, was fixed on a smooth glass surface with glue, and the adhesive sheet was attached to the bottom of the plastic cover with glue. The tablets were suspended directly above the mucous membrane to maintain a balance with the tray on the left side. Immediately, the adhesive sheet was brought into contact with the mucosal tissue and pressed onto the plastic cover with a 5 g counterweight for a period of time. Then, the weight was removed and water was added to another measuring tray at a uniform speed (approximately 1 mL/s). When the tablet was separated from the mucosal tissue, water was added immediately and the added water was weighed to obtain the adhesive force (g) (n = 3).

### 3.10. Ex Vivo Mucoadhesive Time

The time required for tablets to detach from the mucosa under specific experimental conditions was defined as the in vitro mucoadhesive time [55]. Mucosal tissue (1 × 1 cm), which had been immersed in pH 6.8 buffer solution for 1 h, was fixed with glue to the side wall near the bottom of the dissolution medium vessel. The tablets pre-wetted with 1 mL of pH 6.8 buffer solution were placed onto the immobilized mucosa, followed by placing a 5 g weight on top of the tablets and maintaining this pressure for 1 min. Then, 250 mL of pH 6.8 solution heated to 37 °C was added. The time taken for the tablet to detach from the mucosa at 37 ± 0.5 °C and 50 rpm (n = 3) was recorded.

### 3.11. HPLC Analysis

The quantitative analysis of IND (Waters, Milford, MA, USA) was performed using an HPLC detector. The mobile phase consisted of acetonitrile and 0.1 mol/L glacial acetic acid in a volume ratio of 50:50 (*v*/*v*). The flow rate was set to 1 mL/min and the injection volume was 10 μL. The detector was set at 228 nm and the temperature of the analytical column (C18, 150 × 4.6 mm, 5 μm, Phenomenex, Torrance, CA, USA) was controlled at 40 °C. The developed HPLC method was systematically validated in terms of accuracy, linearity, precision, limit of detection (LOD), and limit of quantification (LOQ). The results demonstrated a good linear relationship between drug peak area and concentration, with a correlation coefficient (R^2^) greater than 0.9998, indicating excellent linearity within the investigated concentration range (0.5–100 μg/mL). The accuracy test showed an average recovery between 95% and 105%, confirming that the method is accurate and reliable. In the precision test, the relative standard deviations (RSDs) for both repeatability and intermediate precision were less than 2.0%, demonstrating good method reproducibility. Meanwhile, the LOD and LOQ were determined to be 0.1 μg/mL and 0.3 μg/mL, respectively, which are sufficiently low, meeting the sensitivity requirements for content determination. Overall, the results indicate that the established HPLC method is accurate, sensitive, and robust, and is suitable for the quality evaluation of this formulation. All validation parameters were within acceptable ranges, making the method applicable for the quantitative determination of the drug in tablets and for in vitro dissolution studies (n = 3).

### 3.12. Fourier Transform Infrared Spectroscopy (FTIR)

Pure IND, IND-PM, and IND-SDs were characterized by FTIR spectrometry (FOLI 10-R, INSA Optics Instruments (Shanghai) Ltd., Shanghai, China). The samples were scanned within the wavelength range of 400–4000 cm^−1^ with a resolution of 2 cm^−1^.

### 3.13. Field-Emission Scanning Electron Microscopy (FE-SEM)

The morphologies of the pure IND, IND-SDs, and IND-PM were characterized using an FE-SEM (JSM-7610F, JEOL Ltd., Tokyo, Japan) at an accelerating voltage of 5 kV. Before measurement, the sample was placed on a conductive adhesive and gold plating was performed.

### 3.14. Powder X-Ray Diffraction (PXRD)

PXRD analyses of pure IND, IND-PM, and IND-SDs were performed using a powder X-ray diffractometer (D8, Bruker AXS, Karlsruhe, Germany). The samples were scanned over a 2θ range of 5° to 50° at a scanning rate of 10°/min.

### 3.15. Data Processing and Statistical Analysis

All experiments were performed in triplicate (n = 3). Data were expressed as mean ± standard deviation (SD), with SD represented as error bars in the figures. Statistical comparisons were carried out by one-way analysis of variance (ANOVA). A *p*-value < 0.05 was considered statistically significant; *p* < 0.01 was considered highly statistically significant; *p* < 0.001 was considered extremely statistically significant; and *p* > 0.05 was considered not statistically significant.

## 4. Conclusions

The drug release of orally controlled release tablets is affected by many factors, but the gel performance of polymer is a direct and easy-to-control factor that affects the initial dissolution rate and final release amount. In addition to considering the inherent viscosity and swelling/erosion characteristics of the polymer, its ability to maintain tablet integrity during the dynamic dissolution process should be thoroughly evaluated. This facilitates further regulation of drug release in the middle and late stages, prevents the occurrence of burst release, and simultaneously achieves a dynamic balance between tablet release behavior and mucoadhesive performance. Therefore, to achieve more accurate drug release regulation in the development of oral drug delivery controlled-release solid preparations, the degree of entanglement, viscosity, amorphous region, and other factors related to the gel performance of all gel performance ingredients should be investigated, which can effectively avoid and obviously improve the phenomenon of unstable release rate at the initial stage of drug release.

## Figures and Tables

**Table 1:** Solubility of IND in deionized water, different pH buffers and 1% *w*/*v* aqueous solutions of various excipients (n = 3).

No.	Media	Role	Solubility (μg/mL)	pH Before Equilibration	pH After Equilibration
1	Deionized water	Media	8.8 ± 0.01	5.68 ± 0.01	4.76 ± 0.01
2	pH 1.2	Media	—	1.2 ± 0.01	1.2 ± 0.01
3	pH 6.8	Media	176 ± 0.01	6.8 ± 0.01	6.92 ± 0.01
4	PVP K30	Carrier	6.35 ± 0.01	4 ± 0.01	3.9 ± 0.01
5	PEG 4000	Carrier	39.59 ± 0.01	5.21 ± 0.01	4.97 ± 0.01
6	PEG 6000	Carrier	15.38 ± 0.02	5.1 ± 0.01	4.68 ± 0.01
7	POX 188	Carrier	22.24 ± 0.01	6.05 ± 0.01	4.95 ± 0.01
8	POX 407	Carrier	75.33 ± 0.01	6.03 ± 0.01	4.8 ± 0.01
9	SA	Carrier	79.5 ± 0.01	—	—
10	Tween-80	Surfactant	221.1 ± 0.01	6.05 ± 0.01	5.35 ± 0.01
11	CTAB	Surfactant	598 ± 0.05	4.64 ± 0.01	2.93 ± 0.01
12	SLS	Surfactant	308 ± 0.05	8.78 ± 0.01	6.2 ± 0.01

— IND undergoes hydrolysis in pH 1.2 medium, resulting in its solubility being undetectable, and the SA aqueous solution has a high viscosity and cannot accurately measure the pH value.

**Table 2:** Physical and chemical properties of bilayer tablets (n = 3).

Form.	Weight (mg)	Hardness (N)	Thickness (mm)	Diameter (mm)	Friability (%)	Surface pH	Drug Content (%)	Disintegration Time (min)	Residence Time (h)	Mucoadhesive Strength (g)
F41	130 ± 0.52	49.91 ± 0.6	2.5 ± 0.01	9.8	0.4 ± 0.1	6.8 ± 0.1	100.5 ± 0.1	1200 ± 5.0	8.3 ± 0.1	57 ± 0.13
F42	130 ± 0.23	49 ± 0.4	2.5 ± 0.01	9.8	0.5 ± 0.1	6.79 ± 0.2	98.95 ± 0.2	960 ± 2.5	7.5 ± 0.15	53 ± 0.54
F43	130 ± 0.51	49.94 ± 0.2	2.5 ± 0.02	9.8	0.5 ± 0.01	6.74 ± 0.1	100.5 ± 0.2	120 ± 2.05	0.1 ± 0.24	14 ± 0.45
F44	130 ± 0.43	49.87 ± 0.9	2.5 ± 0.01	9.8	0.5 ± 0.01	6.78 ± 0.1	101.2 ± 0.2	152 ± 2.40	0.15 ± 0.15	18 ± 0.78
F45	130 ± 0.55	49.48 ± 0.2	2.5 ± 0.01	9.8	0.3 ± 0.01	6.72 ± 0.2	99.5 ± 0.2	600 ± 3.5	3.3 ± 0.15	52 ± 0.24

F41: HPMC 100,000; F42: HPMC 4000; F43: PEO 100,000; F44: PEO 1,000,000; F45: carbomer.

**Table 3:** Swelling rate and erosion rate of bilayer tablets (n = 3).

	Time (min)	30	60	90	120	180	240	360	480	600	720	1200
Formulation												
F41	*WU* (%)	998 ± 10	1098 ± 15	1145 ± 14	1249 ± 10	1399 ± 10	1364 ± 10	1456 ± 10	1765 ± 10	1943 ± 10	2300 ± 10	2466 ± 16
F42		970 ± 10	1060 ± 15	1103 ± 10	1187 ± 10	1245 ± 10	1295 ± 11	1358 ± 10	1385 ± 11	1427 ± 15	1717 ± 12	—
F43		890 ± 10	1103 ± 10	1432 ± 14	1871 ± 18	—	—	—	—		—	—
F44		1041 ± 12	1307 ± 12	1670 ± 15	2340 ± 19	—	—	—	—		—	—
F45		989 ± 5	1103 ± 9	1368 ± 10	1719 ± 10	2741 ± 9	3971 ± 9	4343 ± 10	4625 ± 18	8634 ± 20	—	—
F41	*ES* (%)	45 ± 1	59 ± 2	64 ± 1	69 ± 2	72 ± 2	74 ± 2	76 ± 2	80 ± 2	83 ± 2	88 ± 2	94 ± 1
F42		39 ± 2	52 ± 2	58 ± 2	63 ± 2	67 ± 2	68 ± 2	70 ± 2	75 ± 2	80 ± 2	84 ± 1	—
F43		59 ± 2	70 ± 2	84 ± 2	84 ± 2	—	—	—	—		—	—
F44		43 ± 2	67 ± 2	78 ± 1	88 ± 2	—	—	—	—		—	—
F45		34 ± 2	40 ± 2	42 ± 2	44 ± 2	44 ± 4	45 ± 2	46 ± 2	46 ± 3	72 ± 2	—	—

F41: HPMC 100,000; F42: HPMC 4000; F43: PEO 100,000; F44: PEO 1,000,000; F45: Carbomer.

**Table 4:** Formulation compositions of IND-SDs (units: mg).

Composition	Role	F1	F2	F3	F4	F5	F6	F7	F8	F9	F10	F11
IND	Drug	25	25	25	25	25	25	25	25	25	25	25
SA	Carrier	25	—	—	—	—	—	12.5	12.5	12.5	12.5	12.5
PVP K30	Carrier	—	25		—	—	—	12.5	—	—	—	—
POX 188	Carrier	—	—	25	—	—	—	—	12.5	—	—	—
POX 407	Carrier	—	—	—	25	—	—	—	—	12.5	—	—
PEG 4000	Carrier	—	—	—	—	25	—	—	—	—	12.5	—
PEG 6000	Carrier	—	—	—	—	—	25	—	—	—	—	12.5

**Table 5:** Formulation compositions of IR layers (units: mg).

Composition	Role	F12	F13	F14	F15	F16	F17	F18	F19	F20	F21	F22	F23	F24	F25	F26
F1	SDs	25	25	25	25	25	—	—	—	—	—	—	—	—	—	—
F2	SDs	—	—	—	—	—	25	25	25	25	25	—	—	—	—	—
F7	SDs	—	—	—	—	—	—	—	—	—	—	25	25	25	25	25
PVPP	Disintegrants	10	—	—	—	—	10	—	—	—	—	10	—	—	—	—
CMA-Na	Disintegrants	—	10	—	—	—	—	10	—	—	—	—	10	—	—	—
CMS-Na	Disintegrants	—	—	10	—	—	—	—	10	—	—	—	—	10	—	—
L-HPC	Disintegrants	—	—	—	10	—	—	—	—	10	—	—	—	—	10	—
VA64	Disintegrants	—	—	—	—	—	—	—	—	—	10	—	—	—	—	10
MCC	Filler	18	18	18	18	18	18	18	18	18	18	18	18	18	18	18
Mgst	Lubricant	2	2	2	2	2	2	2	2	2	2	2	2	2	2	2
Total weight	—	55	55	55	55	55	55	55	55	55	55	55	55	55	55	55

**Table 6:** Formulation compositions of SR layers (units: mg).

Composition	Role	F27	F28	F29	F30	F31	F32	F33	F34	F35	F36	F37	F38	F39	F40
F2	SDs	25	25	25	25	25	25	25	25	25	—	—	—	—	—
F7	SDs	—	—	—	—	—	—	—	—	—	25	25	25	25	25
PVPP	Disintegrants	10	10	10	10	10	10	10	10	10	10	10	10	10	10
MCC	Filler	18	18	18	18	18	18	18	18	18	18	18	18	18	18
Mgst	Lubricant	2	2	2	2	2	2	2	2	2	2	2	2	2	2
HPMC10,000	Retarding polymer	20	—	—	—	—	—	—	—	—	20	—	—	—	—
HPMC 4000	Retarding polymer	—	20	—	—	—	30	40	50	60	—	20	—	—	—
PEO 100,000	Retarding polymer	—	—	20	—	—	—	—	—	—	—	—	20	—	—
PEO1,000,000	Retarding polymer	—	—	—	20	—	—	—	—	—	—	—	—	20	—
Carbomer	Retarding polymer	—	—	—	—	20	—	—	—	—	—	—	—	—	20
Total weight	—	75	75	75	75	75	85	95	105	115	75	75	75	75	75

**Table 7:** Formulation compositions of IND bilayer tablets (units: mg).

Composition	F41	F42	F43	F44	F45	F46	F47	F48	F49
IR	F17	F17	F17	F17	F17	F17	F17	F17	F17
SR	F27	F28	F29	F30	F31	F32	F33	F34	F35
Total weight	130	130	130	130	130	140	150	160	170

## Data Availability

The original contributions presented in this study are included in the article. Further inquiries can be directed to the corresponding author.

## Abbreviations

The following abbreviations are used in this manuscript:

IND	Indomethacin
SDs	Solid dispersions
PM	Physical mixture
NASID	Non-steroidal anti-inflammatory drug
BCS	Biopharmaceutical Classification System
SEDDS	Self-emulsifying drug delivery systems
IR	Immediate release
SR	Sustained release
PVP K30	Polyvinylpyrrolidone K30
PEO	Polyethylene oxide
HPMC	Hydroxypropyl methylcellulose
POX	Poloxamer
PEG	Polyethylene glycol
SA	Sodium alginate
HPLC	High-performance liquid chromatography
FTIR	Fourier transform infrared spectroscopy
PXRD	Powder X-ray diffraction
FE-SEM	Field-emission scanning electron microscopy
PVPP	Polyvinylpolypyrrolidone
CMC-Na	Sodium carboxymethyl cellulose
CMS-Na	Sodium carboxymethyl starch
L-HPC	Low-substituted hydroxypropyl cellulose
VA64	Copovidone
MCC	Microcrystalline cellulose
Mgst	Magnesium stearate
SLS	Sodium lauryl sulfate
CTAB	Cetyltrimethylammonium bromide
CMC	Critical micelle concentration
